# DEMQOL and DEMQOL-Proxy: a Rasch analysis among those diagnosed with dementia

**DOI:** 10.1186/s12955-019-1216-8

**Published:** 2019-10-26

**Authors:** A. A. Jolijn Hendriks, Sarah C. Smith, Nick Black

**Affiliations:** Department of Health Services Research and Policy, London School of Hygiene & Tropical Medicine, 15-17 Tavistock Place, London, WC1H 9SH, UK

**Keywords:** DEMQOL, DEMQOL-Proxy, Item analysis, Rasch measurement theory

## Abstract

**Background:**

In previous work we concluded that DEMQOL and DEMQOL-Proxy can provide robust measurement of HRQL in dementia when scores are derived from analysis using the Rasch model. As the study sample included people with mild cognitive impairment, we undertook a replication study in the subsample with a diagnosis of dementia (PWD). PWD constitute the population for whom DEMQOL and DEMQOL-Proxy were originally developed.

**Methods:**

We conducted a Rasch model analysis using the RUMM2030 software to re-evaluate DEMQOL (441 PWD) and DEMQOL-Proxy (342 family carers). We evaluated scale to sample targeting, ordering of item thresholds, item fit to the model, and differential item functioning (sex, age, severity, relationship), local independence, unidimensionality and reliability.

**Results:**

For both DEMQOL and DEMQOL-Proxy, results were highly similar to the results in the original sample. We found the same problems with content and response options.

**Conclusions:**

DEMQOL and DEMQOL-Proxy can provide robust measurement of HRQL in people with a diagnosis of dementia when scores are derived from analysis using the Rasch model. As in the wider sample, the problems identified with content and response options require qualitative investigation in order to improve the scoring of DEMQOL and DEMQOL-Proxy.

## Background

DEMQOL and DEMQOL-Proxy [1–3] are disease-specific patient reported outcome measures (PROMs) for measuring health-related quality of life (HRQL) in people with dementia (PWD). Total scores on DEMQOL and DEMQOL-Proxy are typically used as outcomes in intervention and other evaluative studies [4, 5] or, as a measure of disease specific utility [6], in cost-effectiveness studies [7, 8]. In addition, there is growing interest in using PROMs for routine monitoring of the quality of health and social care [9–13], including dementia care [12, 13]. All these purposes require measurements that use an interval scale (i.e. with equal distances between scale points) and, if comparisons use data for individuals (patients), then individual-level standard errors are also required.

Measurements from conventionally developed questionnaires, using the methodology and psychometric principles of classical test theory, do not fulfil these requirements. Though usually treated as interval scores, such scores are de facto ordinal and, in addition, their standard errors are established at the group level, assuming that they are the same for everyone.

In our recent work with people attending a first appointment at Memory Assessment Services [14, 15], we have shown that the scoring for DEMQOL and DEMQOL-Proxy can meet these requirements using modern psychometric methods based on Rasch Measurement Theory [16, 17]. However, the sample in that work was somewhat heterogeneous and included all those referred for suspected dementia irrespective of eventual diagnosis (as that information is not usually available until sometime afterwards). It is possible that the heterogeneous nature of the sample introduced noise to that analysis and the scores generated from that model may not be appropriate for people with a specific diagnosis of dementia. At 6 months follow up, about half of the participants had a confirmed diagnosis of dementia [18]. As DEMQOL/DEMQOL-Proxy were originally designed and validated for use with people with a diagnosis of dementia [1–3], our aim in this paper was to use Rasch Measurement Theory to undertake a diagnostic analysis of the items within DEMQOL and DEMQOL-Proxy to determine if our improved scoring of DEMQOL/DEMQOL-Proxy is replicated in a sample with a confirmed diagnosis of dementia. As these characteristics will potentially vary with each model we wanted to identify if these differed substantially for a model with a dementia diagnosis sample. Together with our original analysis this gives us a more complete diagnostic picture with which to understand how the DEMQOL and DEMQOL-Proxy scales are working and how they can be improved. In particular we investigated whether in this sub-sample, the items of DEMQOL and DEMQOL-Proxy work together as a scale, whether the scale works in the same way for different groups of people, such as men vs women (differential item functioning or DIF), and to what extent PWD are reliably distinguished in terms of their HRQL scores. In addition, the analysis aimed to identify whether anomalies identified in the original analyses such as response options not working as intended and item response dependencies were also found in this sub-sample.

## Methods

### Sample

From the original sample of 1434 people with cognitive impairment and 1030 informal family carers who were attending one of 78 Memory Assessment Services (MAS) for a first referral (either at the clinic or at a home visit) we selected those first attenders who were available at 6 months follow up and had a diagnosis of dementia, and their family carers (if present). For pragmatic reasons, participants who were diagnosed after 6 months were not included.

### Instruments

DEMQOL consists of 28 questions and DEMQOL-Proxy consists of 31 questions, each assessed on a 4-point Likert-type response scale: *a lot*, *quite a bit*, *a little*, *not at all*. The questions were derived from five conceptual domains: health and well-being, cognitive functioning, daily activities, social relationships and self-concept [2]. Separate sub-scales are not supported so both instruments are scored as a single overall score. Emotion items have the stem “Have you felt…”, all other items have the stem “How worried have you been about…”. There is also an additional overall quality of life question, answered on a 4-point scale: *very good*, *good*, *fair*, *poor*. The items are scored according to a standard scoring algorithm [19] to produce an overall score where higher scores represent better HRQL. See Smith et al. [1–3] for details on the development and validation of DEMQOL and DEMQOL-Proxy based on classical test theory. DEMQOL is self-reported by the PWD (though interviewer-administered) and is appropriate for use in mild to moderate dementia. DEMQOL-Proxy is proxy-reported by a family carer on behalf of the PWD, either self-administered [20] or interviewer-administered, and can be used at all stages of dementia. The two instruments are intended to be used together. DEMQOL has been shown to have reliability (internal consistency and test-retest) and validity (convergent and discriminant) in mild/moderate dementia. DEMQOL-Proxy has been shown to have reliability (internal consistency and test-retest) and validity (convergent and discriminant) in mild/moderate and severe dementia [1, 3] Disease-specific utility scores are also available for both instruments [6] The robustness of both instruments has also been shown to be improved by using a scoring algorithm based on Rasch Measurement Theory [14].

### Data analysis

We conducted psychometric analyses using the Rasch model (in RUMM2030 software [21]), separately for DEMQOL and DEMQOL-Proxy. For all analyses we used the partial credit model (although all the items have the same 4-point Likert type scale). This was because of the diagnostic nature of the analyses which included an evaluation of whether each responses scale was actually used in a similar way.

As in our original study [14], we investigated: scale to sample targeting, how well the items work together as a measuring instrument (ordering of item response thresholds, item fit, item dependency, differential item functioning by sex, age group, severity or relationship, on the basis that DEMQOL/DEMOQL-Proxy include a range of items about different aspects of daily life which arguably could also be affected by the aging process itself, gender roles and expectations and the deteriorating nature of dementia where eventually patients lose insight about their condition) and how well the instrument measures the people in the sample (person separation index, PSI). See the original study for details on the analyses. The positive emotion items were excluded from the analysis as in both this data set and our previous datasets [14, 15] they appear to be trait-like rather than state-like items and are thus qualitatively different from the rest of the instrument. We therefore focussed our analyses on the smaller remaining set of 23 items for DEMQOL and 26 items for DEMQOL-Proxy. Family wise *p* values were set at 0.01 for item fit and the more conservative value of 0.05 for DIF (to accommodate main effect class interval, main effect person factor and their interaction). For individual tests at the item level these were Bonferroni corrected within the RUMM2030 software. Therefore, at the item level *p* values for item fit were *p* = 0.000435 (DEMQOL, 23 items) and *p* = 0.000385 (DEMQOL-Proxy, 26 items), and for DIF *p* = 0.000725 (DEMQOL, 69 comparisons) and *p* = 0.000641 (DEMQOL-Proxy, 78 comparisons).

## Results

### Descriptive characteristics of the sample

The sample consisted of 441 PWD, 204 males and 237 (53.7%) females with a diagnosis of dementia and a completed questionnaire. Their age ranged from 58 to 96 years (mean age = 79.6, *SD* = 6.8). In addition, we had data for 342 family carers, 110 males and 232 (67.8%) females. Carers’ age ranged from 31 to 91 years (mean age = 67.5, *SD* = 12.7). They were mostly the spouse (63.1%), or son or daughter (27.7%) of the PWD. Table 1 shows further details of the sample. The sample is demographically very similar to the original sample with a few slight differences; participants are slightly more likely to be female, older and less deprived. Also, their carers tend to be slightly older and are slightly more likely to be living with the person with dementia.

**Table 1:** Demographic characteristics of PWD and carers

	Subsample dementia	Full sample
Characteristics	*n* (%)	*n* (%)
PWD		
Sex		
Male	204 (46.3)	682 (47.8)
Female	237 (53.7)	746 (52.2)
Age		
< 73	68 (15.4)	352 (24.6)
73–78	102 (23.1)	334 (23.4)
79–83	141 (32.0)	352 (24.6)
> 83	130 (29.5)	390 (27.3)
Ethnicity		
White/White British	415 (94.5)	1343 (94.0)
Other ethnicity	24 (5.5)	78 (5.5)
Missing	2	7
Deprivation quintiles^a^		
1 – least deprived	125 (28.9)	349 (24.9)
2	96 (22.2)	299 (21.4)
3	78 (18.0)	280 (20.0)
4	70 (16.2)	253 (18.1)
5 – most deprived	64 (14.8)	219 (15.6)
Missing	8	28
Number of comorbidities^b^		
0	96 (21.8)	315 (22.1)
1	121 (27.4)	376 (26.3)
2	107 (24.3)	332 (23.2)
3	71 (16.1)	232 (16.2)
4 or more	46 (10.4)	173 (12.2)
Missing	0	6
Carers:		
Sex		
Male	110 (32.2)	312 (30.5)
Female	232 (67.8)	710 (69.5)
Age (y)		
< 57	75 (21.9)	245 (24.0)
57–67	74 (21.6)	254 (24.9)
68–76	90 (26.3)	272 (26.6)
> 76	103 (30.1)	251 (24.6)
Ethnicity		
White/White British	323 (95.6)	958 (95.2)
Other ethnicity	15 (4.4)	48 (4.8)
Missing	4	16
Relationship		
Husband/wife/partner	214 (63.1)	615 (61.0)
Son/daughter	94 (27.7)	295 (29.2)
Son/daughter-in-law	11 (3.2)	25 (2.5)
Sibling	6 (1.8)	14 (1.4)
Other relative	6 (1.8)	28 (2.8)
Friend	3 (0.9)	16 (1.6)
Neighbour	3 (0.9)	7 (0.7)
Other	2 (0.6)	9 (0.9)
Missing	3	13
Living with relative/friend		
Yes	237 (70.3)	683 (68.0)
No	100 (29.7)	321 (32.0)
Missing	5	18

*PWD* people with dementia

^a^On the basis of the Index of Multiple Deprivation 2010 score. The Index of Multiple Deprivation ranks deprivation in quintiles based on patients’ residential postcodes and is used as an indicator of socioeconomic status

^b^Selected from the following list of chronic conditions: heart disease (e.g. angina, heart attack or heart failure), high blood pressure, problems caused by stroke, leg pain when walking due to poor circulation, lung disease (e.g. asthma, chronic bronchitis or emphysema), diabetes, kidney disease, disease of the nervous system (e.g. Parkinson’s disease or multiple sclerosis), liver disease, cancer (within the last 5 years), depression or arthritis

### Targeting

**Fig. 1:**
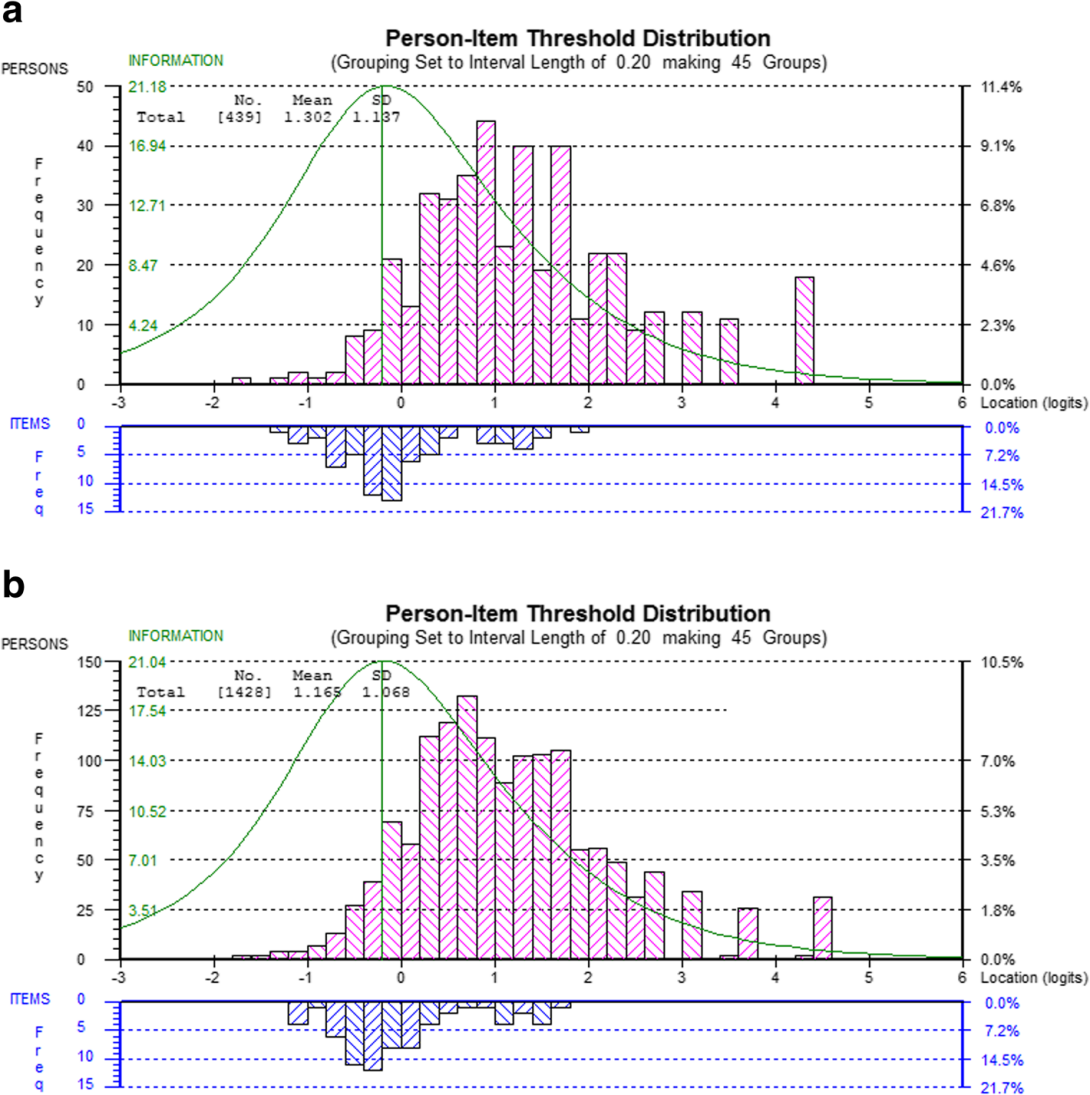
Person-item threshold distribution for DEMQOL (23 items) in the subsample of people with dementia (**a**) and in the full sample (**b**)

**Fig. 2:**
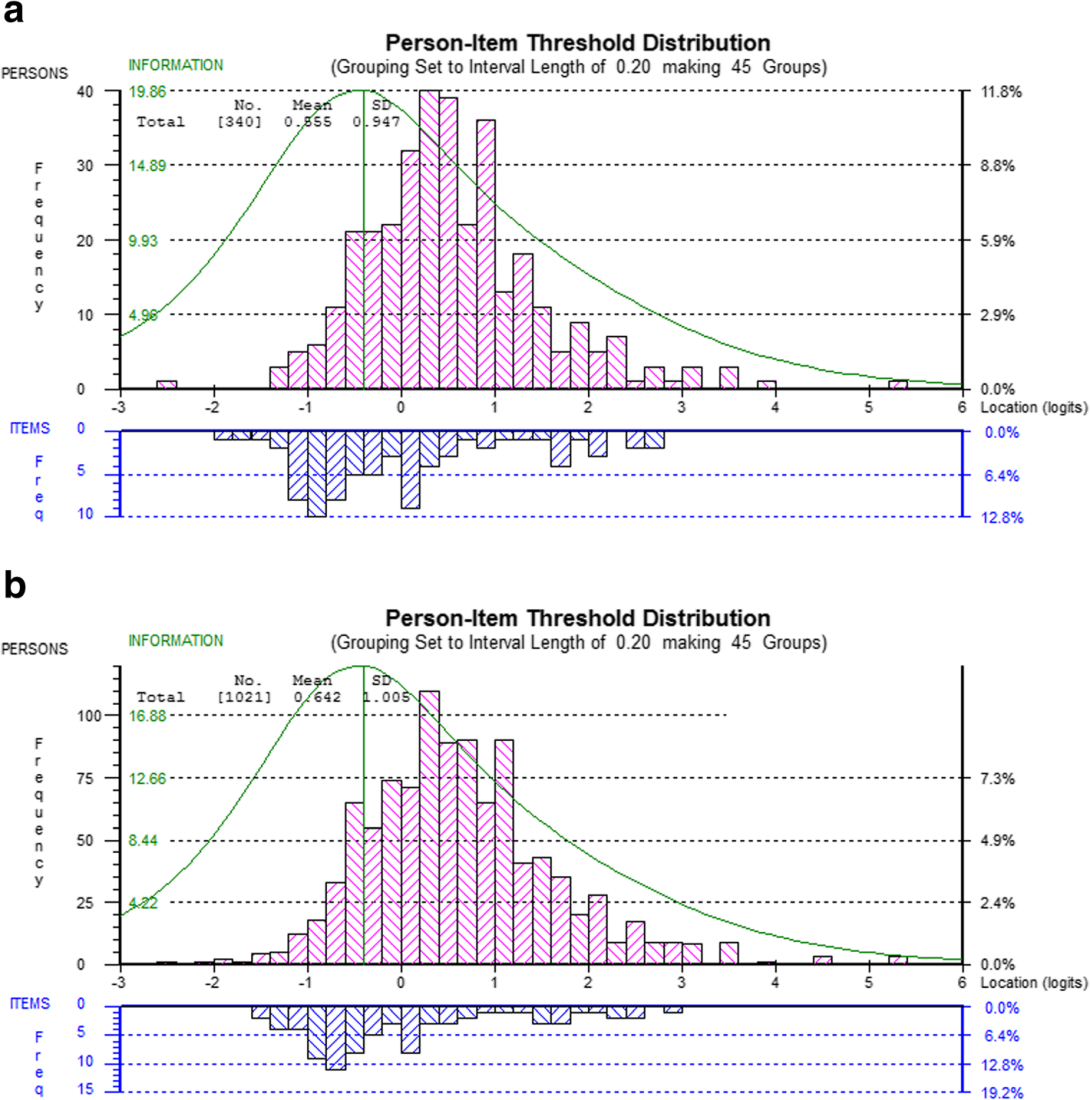
Person-item threshold distribution for DEMQOL-Proxy (26 items) in the subsample of people with dementia (**a**) and in the full sample (**b**)

For both DEMQOL (23 items) (Fig. 1) and DEMQOL-Proxy (26 items) (Fig. 2) the targeting was very similar to the targeting in the original, full, sample of first attenders to MAS. In this subsample, DEMQOL item threshold locations ranged from roughly − 1.4 to + 2.0 logits and person locations from roughly − 1.8 to + 4.4 logits, compared with − 1.2 to + 1.8 logits and − 1.8 to + 4.6 logits, respectively, in the full sample. As before, there was a lack of item thresholds at the high end of the continuum. In this subsample, DEMQOL-Proxy item threshold locations ranged from roughly − 2.0 to + 2.8 logits and person locations from roughly − 2.6 to + 5.4 logits, compared with − 1.6 to + 3.0 logits and − 2.6 to + 5.4 logits, respectively, in the full sample. As in the full sample, DEMQOL-Proxy showed less of a gap in item thresholds at the high end of the continuum than DEMQOL because in contrast to DEMQOL it is not just positive emotion items having the highest located item thresholds.

### Ordering of item thresholds

Seven of the 23 DEMQOL items and four of the 26 DEMQOL-Proxy items showed disordered thresholds, compared with five for DEMQOL and three for DEMQOL-Proxy in the previous full sample. In both cases, we found the same items disordered as in the full sample. The two additional items for DEMQOL were “having felt lonely” and “having been worried about forgetting what day it is”. The one additional item for DEMQOL-Proxy was “having been worried about forgetting where he/she is”. As in the full sample, all disordered thresholds showed that the middle two categories (“quite a bit” and “a little”) were not used as intended.

### Item fit

As in the full sample, none of the 23 DEMQOL items (Table 2) or 26 DEMQOL-Proxy items (Table 3) showed misfit to the model, considering the fit residual, chi square value and ICC together. More specifically, as in the full sample, none of the 23 DEMQOL items and 26 DEMQOL-Proxy items showed statistically significant misfit to the model. Only two of the 23 DEMQOL items (compared with nine in the full sample) and one of the 26 DEMQOL-Proxy items (compared with six in the full sample) showed large fit residuals (> +/− 2.5).

**Table 2:** Diagnostic statistics for DEMQOL (23 items)

	Subsample dementia					Full sample				
Item	Location	Fit Residual	ChiSq	*p*	DIF	Location	Fit Residual	ChiSq	*p*	DIF
2. Worried or anxious	0.331	1.081	5.84	0.44	*ns*	0.349	0.886	3.54	0.94	*ns*
4. Frustrated	0.483	0.338	4.58	0.60	*ns*	0.546	0.257	2.66	0.98	*ns*
7. Sad	0.036	− 0.416	4.23	0.65	*ns*	0.080	−0.255	6.58	0.68	*ns*
8. Lonely	− 0.238	1.948	19.47	0.00	*ns*	−0.206	2.222	5.59	0.78	*ns*
9. Distressed	−0.531	− 0.252	2.31	0.89	*ns*	−0.391	−2.395	7.04	0.63	*ns*
11. Irritable	0.050	0.497	3.16	0.79	*ns*	0.007	0.361	1.66	1.00	*ns*
12. Fed-up	0.489	0.192	3.69	0.72	*ns*	0.425	0.361	2.20	0.99	*ns*
13. Things you wanted but couldn’t	0.735	2.408	21.20	0.00	*ns*	0.829	**4.077**	12.51	0.19	*ns*
14. Forgetting happened recently	0.673	−0.974	11.45	0.08	*ns*	0.654	−1.100	4.43	0.88	*ns*
15. Forgetting who people are	0.018	−0.207	6.34	0.39	*ns*	−0.043	**2.511**	6.68	0.67	*ns*
16. Forgetting what day it is	0.375	1.342	5.28	0.51	*ns*	0.135	**4.339**	11.45	0.25	*ns*
17. Thoughts being muddled	0.280	−1.107	11.45	0.08	*ns*	0.126	−2.376	8.27	0.51	*ns*
18. Difficulty making decisions	−0.123	−1.527	8.23	0.22	*ns*	−0.103	**− 2.777**	8.08	0.53	*ns*
19. Poor concentration	0.305	**−2.557**	16.96	0.01	*ns*	0.298	−2.338	6.86	0.65	*ns*
20. Not having enough company	−0.468	1.750	16.53	0.01	*ns*	−0.415	−0.981	5.01	0.83	*ns*
21. Get on with people close to you	−0.366	−2.134	10.65	0.10	*ns*	−0.422	−1.291	5.21	0.82	*ns*
22. Getting the affection you want	−0.564	−1.112	6.15	0.41	*ns*	−0.525	0.328	6.63	0.68	*ns*
23. People not listening to you	−0.635	0.290	5.56	0.47	*ns*	−0.479	−0.989	5.73	0.77	*ns*
24. Making yourself understood	−0.319	−1.278	6.88	0.33	PWD sex	−0.337	−1.792	4.27	0.89	*ns*
25. Getting help when needed	−0.466	−1.278	11.54	0.07	*ns*	−0.511	**−2.833**	5.07	0.83	*ns*
26. Getting to the toilet in time	−0.202	1.596	16.87	0.01	*ns*	−0.267	**3.543**	17.96	0.04	*ns*
27. How you feel in yourself	0.011	**−2.538**	16.88	0.01	*ns*	0.016	**−4.440**	14.24	0.11	*ns*
28. Overall health	0.125	−0.816	5.74	0.45	*ns*	0.232	−1.848	6.27	0.71	*ns*

*Note*. Fit residuals in bold are outside the acceptable range of +/− 2.5. Location = average item threshold location (logit). ChiSq = chi square value; *p* = chi square probability. DIF = differential item functioning; *ns* = non-significant. None of the chi square tests is statistically significant at familywise α = 0.01 (Bonferroni-corrected: *p* < 0.000435)

**Table 3:** Diagnostic statistics for DEMQOL-Proxy (26 items)

	Subsample dementia					Full sample				
Item	Location	Fit Residual	ChiSq	*p*	DIF	Location	Fit Residual	ChiSq	*p*	DIF
2. Worried or anxious	0.456	− 0.264	6.83	0.34	*ns*	0.637	0.278	3.76	0.93	*ns*
3. Frustrated	0.502	− 0.052	4.28	0.64	*ns*	0.574	−0.677	4.27	0.89	*ns*
5. Sad	− 0.464	− 0.175	13.58	0.03	*ns*	−0.330	−1.455	7.01	0.64	*ns*
7. Distressed	−0.532	−1.198	6.24	0.40	*ns*	−0.425	−2.095	6.48	0.69	*ns*
9. Irritable	0.000	0.985	14.32	0.03	PWD sex	0.052	0.919	3.07	0.96	PWD sex PWD age Relation
10. Fed-up	0.216	−0.266	13.69	0.03	*ns*	0.287	−1.249	3.05	0.96	*ns*
12. Memory in general	0.623	1.094	4.72	0.58	*ns*	0.664	1.529	6.49	0.69	*ns*
13. Forgetting that happened long ago	−0.610	**2.793**	12.90	0.04	*ns*	−0.467	**4.704**	19.16	0.02	*ns*
14. Forgetting that happened recently	1.507	−0.172	5.16	0.52	*ns*	1.462	0.373	4.66	0.86	*ns*
15. Forgetting people’s names	0.755	0.478	2.61	0.86	*ns*	0.684	**2.627**	8.42	0.49	*ns*
16. Forgetting where he/she is	−0.754	0.399	9.51	0.15	*ns*	−0.866	−0.539	5.87	0.75	*ns*
17. Forgetting what day it is	0.632	1.541	2.52	0.87	*ns*	0.451	2.045	4.86	0.85	Severity
18. Thoughts being muddled	0.732	−2.251	21.98	0.00	Carer age Relation	0.604	**−3.813**	25.07	0.00	*ns*
19. Difficulty making decisions	0.535	−1.790	17.28	0.01	*ns*	0.458	**−3.475**	15.15	0.09	*ns*
20. Making him/herself understood	−0.253	0.748	13.65	0.03	*ns*	−0.201	2.456	3.60	0.94	*ns*
21. Keeping him/herself clean	−0.685	2.373	17.93	0.01	*ns*	−0.744	2.423	7.96	0.54	*ns*
22. Keeping him/herself looking nice	−0.746	1.470	17.92	0.01	*ns*	−0.702	2.008	9.74	0.37	*ns*
23. Getting from the shops	−0.436	−0.795	3.45	0.75	*ns*	−0.501	−0.922	3.61	0.94	*ns*
24. Using money to pay	−0.411	−0.573	4.80	0.57	*ns*	−0.578	−1.365	5.59	0.78	*ns*
25. Looking after finances	−0.161	1.300	7.54	0.27	*ns*	−0.304	0.915	9.25	0.41	*ns*
26. Things taking longer	0.378	−1.195	8.67	0.19	*ns*	0.402	**−2.987**	13.39	0.15	*ns*
27. Getting in touch with people	−0.376	−0.843	7.23	0.30	*ns*	−0.472	−1.684	6.39	0.70	*ns*
28. Not having enough company	−0.446	1.012	6.61	0.36	PWD sex PWD age Carer age Relation	−0.352	2.197	7.79	0.56	PWD sex Carer age Relation
29. Not being able to help other people	−0.542	0.012	4.29	0.64	*ns*	−0.472	1.136	5.80	0.76	*ns*
30. Not playing a useful part	−0.281	−0.383	7.64	0.27	*ns*	−0.231	−2.067	8.27	0.51	*ns*
31. His/her physical health	0.360	0.783	10.10	0.12	*ns*	0.369	**2.762**	9.22	0.42	*ns*

*Note*. Fit residuals in bold are outside the acceptable range of +/− 2.5. Location = average item threshold location (logit). ChiSq = chi square value; *p* = chi square probability. DIF = differential item functioning; *ns* = non-significant. None of the chi square tests is statistically significant at α = 0.01 (Bonferroni-corrected: *p* < 0.000385)

### Differential item functioning

None of the 23 DEMQOL items showed DIF for PWD age group or severity, which is in agreement with the findings in the full sample. However, one of the 23 DEMQOL items showed uniform DIF for PWD sex: given the same amount of HRQL, females scored higher than males on “worried about making yourself understood” (Table 2). This item showed no DIF in the full sample. Three of the 26 DEMQOL-Proxy items showed uniform DIF, two of them were the same ones as in the full sample (Table 3). As in the full sample, “worried about not having enough company” showed uniform DIF for multiple sources. It showed DIF for PWD sex (carers of female PWD reporting more worry about not having enough company), PWD age group (no clear pattern), carer age group (no clear pattern) and relationship to the PWD (carers who are a spouse reporting less worry about not having enough company than child/other carers). “Felt irritable” showed less sources of uniform DIF than in the full sample. Its only source was PWD sex (carers of male PWD reporting more irritability), not PWD age group or relationship. Differently from the findings in the full sample, “worried about thoughts being muddled” showed uniform DIF for carer age group (older carers reporting less worry for the PWD) and relationship (spouse carers reporting less worry for the PWD than child/other carers). However, “worried about forgetting what day it is” showed no DIF in the subsample of PWD compared with DIF for severity in the full sample. None of the DEMQOL and DEMQOL-Proxy items showed non-uniform DIF. This is in agreement with the findings in the full sample.

### Local Independence

We found one residual correlation > 0.3 for DEMQOL (felt lonely/worried about not having enough company: 0.33), one less than in the full sample. We found 11 residual correlations > 0.3 for DEMQOL-Proxy, of which nine pairs were identical to those (also 11) in the full sample. As in the full sample, item dependency occurred mainly among the negative emotion items, among the cognition items and among the daily activities items of DEMQOL-Proxy. Table 4 (DEMQOL) and Table 5 (DEMQOL-Proxy) show all residual correlations larger than zero and those > 0.3 are highlighted. For both DEMQOL and DEMQOL-Proxy, pattern and strength of the residual correlations strongly resembled those in the full sample.

**Table 4:**
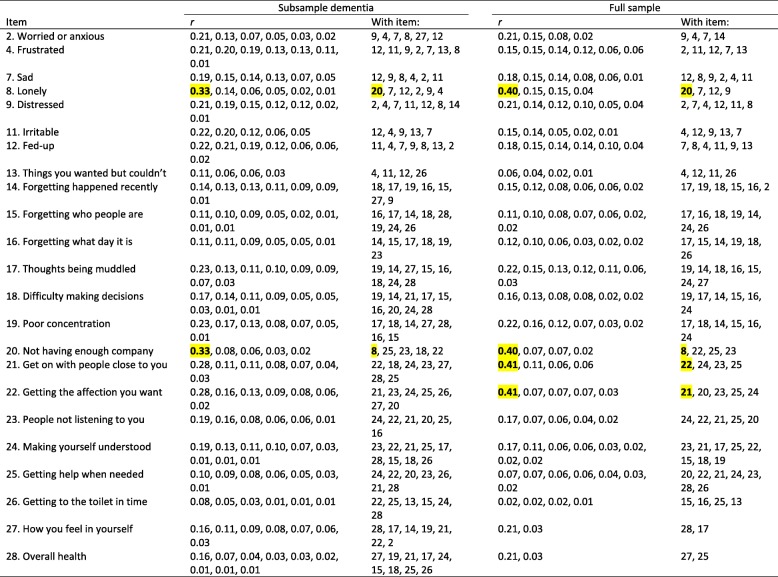
Item residual correlations DEMQOL (23 items)

*Note*. We show the residual correlations at the item level, therefore twice. For instance, a residual correlation of *r* = 0.21 between item 2 and item 9 is also shown as a residual correlation of *r* = 0.21 between item 9 and item 2. Residual correlations > 0.3 are highlighted

**Table 5:**
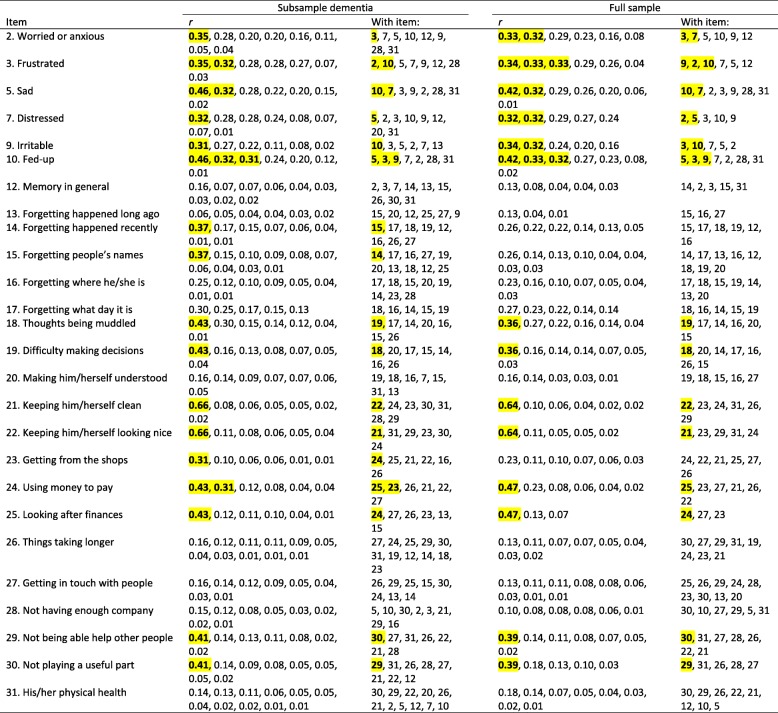
Item residual correlations DEMQOL-Proxy (26 items)

*Note*. We show the residual correlations at the item level, therefore twice. For instance, a residual correlation of *r* = 0.35 between item 2 and item 3 is also shown as a residual correlation of *r* = 0.35 between item 3 and item 2. Residual correlations > 0.3 are highlighted

### Unidimensionality

The 23 DEMQOL items formed an acceptably unidimensional scale though the 26 items in DEMQOL-Proxy were not unidimensional. This is in accordance with our findings in the previous full sample. For DEMQOL the two subsets of measurements based on the four highest and four lowest loading items on the Rasch factor differed significantly for 7.4% [5.2; 10.3] of the cases at the 5% level and for 1.2% [0.4; 3.5] of the cases at the 1% level. These percentages are marginally more than in the full sample (7.1 and 1.1% respectively). For DEMQOL-Proxy, the two subsets of measurements differed significantly for 12.5% [9.4; 16.5] of the cases at the 5% level and for 4.2% [2.1; 8.0] at the 1% level, slightly more than in the full sample (11.9 and 3.0% respectively).

### Reliability

For the 23 DEMQOL items PSI = 0.86 (compared with 0.87 in the full sample), and for the 26 DEMQOL-Proxy items PSI = 0.90 (compared with 0.91 in the full sample). Both these are similar to the findings in the original full sample.

### Overall fit to the model

For both DEMQOL (23 items) and DEMQOL-Proxy (26 items) the overall chi square statistic was significant (both: *p* < 0.001) suggesting that the data did not fit the model. However, for DEMQOL (but not DEMQOL-Proxy, *p* = 0.003) the data did fit the model after rescoring the items with disordered thresholds (DEMQOL: *p* = 0.13).

### Rasch model based (logit) scores and their benefit

**Fig. 3:**
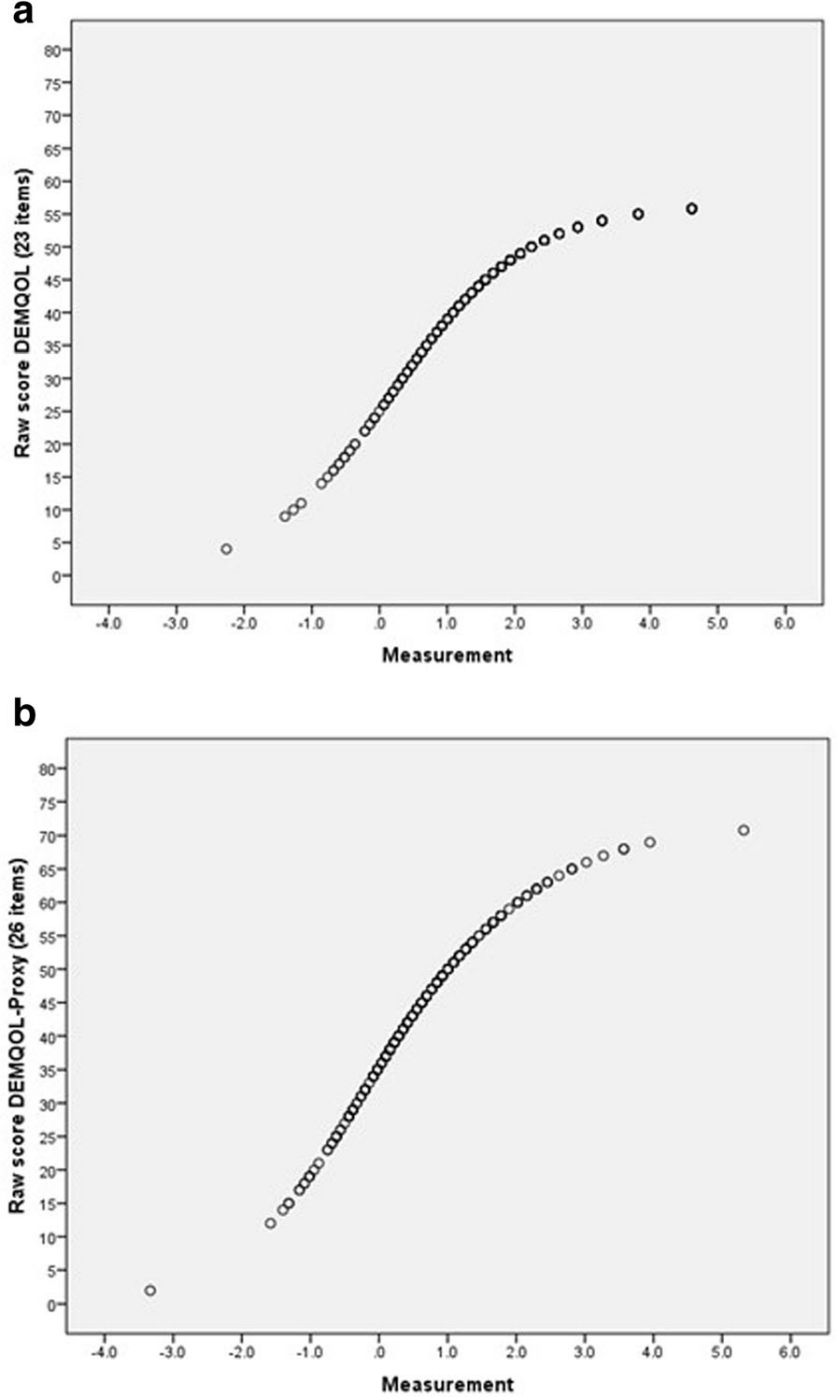
Relationship between raw scores and measurements (logits) for DEMQOL (23 items) (**a**) and DEMQOL-Proxy (26 items) (**b**) in the subsample of people with dementia

In Fig. 3 we show the relationship between raw scores (simple sums of item scores) and measurements based on the Rasch model (logits) for DEMQOL and DEMQOL-Proxy. The S-shaped curve clearly indicates that at the extremes of the distribution there is benefit from deriving the Rasch model based scores. For both DEMQOL (23 items) and DEMQOL-Proxy (26 items), a 10-point increase at one of the extremes of the raw score scale corresponds to a much larger increase in logits than a 10-point increase in the middle of the raw score scale. This strongly resembles what we found in the full sample.

## Discussion

The improved scoring of DEMQOL and DEMQOL-Proxy previously developed in a heterogeneous sample of people with cognitive impairment using Rasch Measurement Theory [17] also holds for the specific subset of people with a diagnosis of dementia, for whom DEMQOL and DEMQOL-Proxy were originally developed. The improved Rasch-model based scores for DEMQOL and DEMQOL-Proxy can provide more robust and meaningful estimates of change than their original scores based on classical test theory [1, 3]. Rasch-model based scores are truly interval measurements and invariant (i.e. independent of the sampling distributions of persons and items in which they were established). As such they are appropriate for use with individual people, such as in decision making about their clinical management. Our previous recommendation that DEMQOL and DEMQOL-Proxy should continue to be administered in their original format (28 and 31 questions respectively) and that the more robust scoring derived from our Rasch based analyses should be used, is also appropriate for the specific sub-sample of people with a dementia diagnosis.

This study identified the same anomalies as the full sample analysis and these need to be addressed. Disordered thresholds indicate that response options are not working as intended. In completing these items, PWD and their family carers make less fine distinctions than the four-category response scale offers. As previously recommended [14], future qualitative work should investigate why this is the case and how the response scale may be improved.

Other anomalies replicated in the present study are item response dependencies and DIF. Item pairs that are dependent share additional variance over and above the variance they share because of measuring the same underlying HRQL construct. Again, in future qualitative work we need to investigate if perhaps these items are not optimally phrased or are redundant. Furthermore, we need to investigate why some of the items show DIF and what we can do about it. Although uniform DIF can be resolved by splitting the affected items (e.g. separate items for male and female PWD), items showing no DIF are to be preferred.

This replication study is limited in much the same ways as our previous analyses [14]. Our data did not allow us to investigate whether the scales are similar across ethnic groups, nor was it possible to investigate any differences across different levels of severity. This analysis has also not addressed any of the issues relating to the relationship between self-reports from DEMQOL and proxy-reports from DEMQOL-Proxy.

## Conclusion

In previous work we concluded that DEMQOL and DEMQOL-Proxy can provide robust measurement of HRQL in dementia when scores are derived from analysis using the Rasch model [14]. The results reported here, are similar enough to our previous findings to indicate that the improved scoring is appropriate for the specific sub-sample with a diagnosis of dementia. Future work should focus on improving content (e.g. the positive emotion items and investigating DIF) and response scales.

## Data Availability

The datasets generated and analysed during the current study are not publicly available because the study is still ongoing, but after the end of the study can be requested from the second author, Dr. Sarah C. Smith, sarah.smith@lshtm.ac.uk.

## Abbreviations

DIF: Differential item functioning
HRQL: Health-related quality of life
MAS: Memory Assessment Services
PROMs: Patient reported outcome measures
PSI: Person Separation Index
PWD: People with dementia
